# Regulation of Blood–Brain Barrier Permeability via JNK Signaling Pathway: Mechanisms and Potential Therapeutic Strategies for Ischemic Stroke, Alzheimer’s Disease and Brain Tumors

**DOI:** 10.3390/molecules30112353

**Published:** 2025-05-28

**Authors:** Mark B. Plotnikov, Anna M. Anishchenko, Andrei I. Khlebnikov, Igor A. Schepetkin

**Affiliations:** 1 Department of Pharmacology, Goldberg Research Institute of Pharmacology and Regenerative Medicine, Tomsk National Research Medical Center, Russian Academy of Sciences, Tomsk 634028, Russia; mbp2001@mail.ru (M.B.P.); nuska-80@mail.ru (A.M.A.); 2 Faculty of Radiophysics, National Research Tomsk State University, Tomsk 634050, Russia; 3 Department of Pharmacology, Siberian State Medical University, Tomsk 634050, Russia; 4 Kizhner Research Center, National Research Tomsk Polytechnic University, Tomsk 634050, Russia; 5 Department of Microbiology and Cell Biology, Montana State University, Bozeman, MT 59717, USA

**Keywords:** Alzheimer’s disease, blood–brain barrier, brain tumor, ischemia/reperfusion, c-Jun N-terminal kinase (JNK), JNK pathway, JNK inhibitor

## Abstract

Disruption of the blood–brain barrier (BBB) accompanies many brain diseases, including stroke, neurodegenerative diseases, and brain tumors, leading to swelling, increased neuroinflammation, and neuronal death. In recent years, it has become clear that the c-Jun N-terminal kinase (JNK) signaling pathway is involved in disruption of the structural integrity of the BBB. Activation of the JNK signaling pathway has a negative effect on the functioning of the cellular elements of the neurovascular unit that form the BBB. The aim of this review is to assess the role of the JNK signaling pathway in the disruption of the structural integrity of the BBB in animal models of stroke (MCAO/R, middle cerebral artery occlusion with reperfusion), Alzheimer’s disease, and brain tumors and to analyze the effects of compounds of various natures that directly or indirectly affect the activity of the JNK signaling pathway. These compounds can reduce damage to the BBB and brain edema, reduce neuroinflammation and oxidative stress, reduce the expression of proapoptotic factors, and increase the expression of tight junction proteins. Certain compounds mitigate BBB dysfunction, being promising candidates for neuroprotective therapies. These agents exert their effects, in part, through inhibition of the c-Jun N-terminal kinase (JNK) signaling pathway, a mechanism linked to reduced neuronal damage and improved BBB integrity.

## 1. Introduction

Disruptions of the blood–brain barrier (BBB) accompany many brain diseases, including stroke, neurodegenerative diseases, brain tumors, traumatic brain injury, and epilepsy, as well as infectious meningitis and encephalitis [1,2,3,4,5,6,7]. According to the World Stroke Organization, ischemic stroke is the leading cause of death and disability worldwide [8]. During cerebral ischemia and largely after reperfusion/reoxygenation, a number of processes are triggered, including oxidative stress and inflammation, leading to disruption of BBB integrity. BBB disruption is a key link in the pathogenesis of ischemic stroke, leading to edema, intracranial hemorrhage, neuroinflammation, and neuronal apoptosis [9,10]. Increased BBB permeability associated with cerebral ischemia/reperfusion (I/R) may contribute to worse outcome after stroke [11].

The most effective strategy for treating ischemic stroke is to eliminate vascular occlusion with thrombolytic agents at the earliest possible stage [12]. Unfortunately, the narrow “time window” for the application of this technology limits their clinical application [13]. Moreover, reperfusion technologies trigger several processes that cause edema, inflammation, and hemorrhagic transformation [14,15]. The ability to reduce hemorrhagic transformation by protecting the BBB from damaging factors is crucial for increasing the efficacy and safety of thrombolytic therapy [11,16].

The various technologies used to treat BBB damage in ischemic stroke include physical approaches, chemical agents, traditional Chinese medicine, neural stem cell therapy, microRNA, and others [17]. In recent years, it has become clear that the c-Jun N-terminal kinase (JNK) signaling pathway is involved in the disruption of the structural integrity of the BBB in ischemic stroke. Compounds of various natures can reduce the severity of BBB damage and cerebral edema by preventing or limiting the involvement of the JNK pathway after I/R [18,19,20,21]. The JNK pathway is involved in BBB damage and other brain diseases, including Alzheimer’s disease (AD), brain tumors, epilepsy, traumatic brain injury, and infectious meningitis [5,22,23,24,25]. However, data on the regulation of BBB permeability through the JNK pathway in epilepsy, meningitis, and encephalitis are either absent or rare [26,27,28].

The aim of this review is to analyze the effectiveness of compounds that directly or indirectly affect the activity of the JNK signaling pathway in regulating BBB permeability in cerebral I/R as a basis for new therapeutic strategies in ischemic stroke. In addition, it was of interest to analyze whether the mechanisms of correction of BBB disorders in ischemic stroke are realized by regulating the activity of the JNK pathway to maintain BBB permeability in other socially significant diseases, such as AD and brain tumors.

## 2. JNK

JNKs belong to a family of mitogen-activated protein kinases (MAPKs). MAPKs are serine/threonine kinases and are important signaling components that link extracellular stimuli to a multitude of intracellular responses that affect cell growth, differentiation, survival, and metabolic regulation [29]. Three major MAPK classes have been reported: extracellular signal-regulated kinases 1/2 (ERK1/2), p38, and JNK [30]. MAPKs are activated in response to various stress stimuli such as UV radiation, heat, oxidative stress, and osmotic shock, and I/R injury of the brain and the heart [31,32,33,34,35,36]. The family of JNK includes 10 isoforms encoded by three genes: *JNK1* (four isoforms), *JNK2* (four isoforms), and *JNK3* (two isoforms) [37,38]. JNK1 and JNK2 are found in all cells and tissues of the body, while JNK3 is expressed mainly in the heart, brain, and testicles [36]. In normal cells, JNKs phosphorylate and regulate the transcription factors c-Jun, activating transcription factor 2 (ATF2), Elk-1, p53, and c-Myc and some non-transcriptional factors such Bim, Bad, and some members of the Bcl-2 interacting mediator (BIM) of cell death family (Bcl-2 and Bcl-X-L) in response to various extracellular stimuli [39,40,41].

JNK pathways have an important role in axon elongation and corticogenesis during embryonic development [42,43] and the formation of new neurons after injury or stress in adults [44]. In addition, JNK are involved in the pathogenesis of many diseases such as stroke, atherosclerosis, AD, and Parkinson’s disease [38,45,46,47]. In vivo studies have demonstrated early and prolonged activation of JNK pathway in the brain after cerebral ischemia [48,49,50,51,52,53,54]. JNK inhibition protects against focal and global ischemic brain injury [46,50,55,56,57].

## 3. BBB

**Figure 1:**
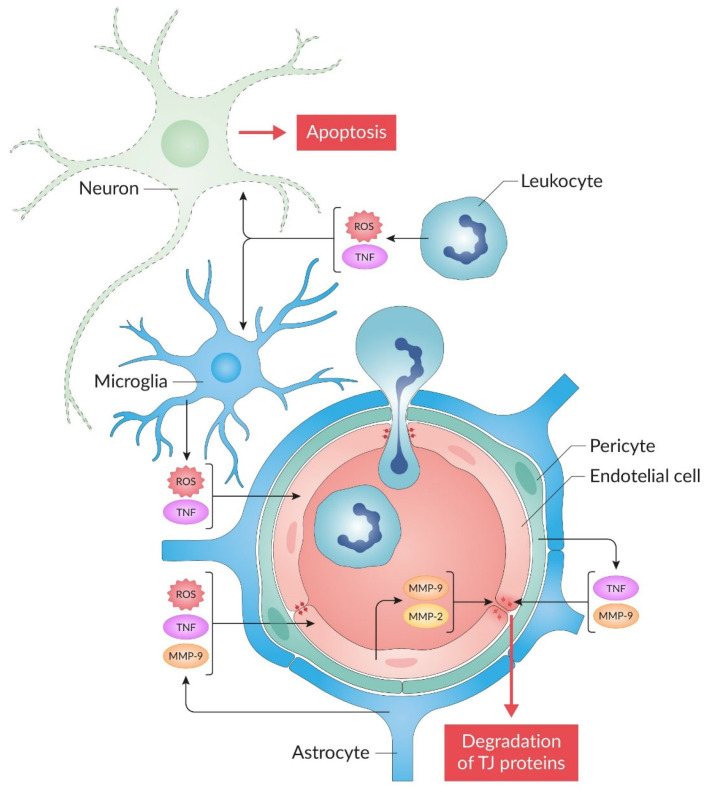
Schematic representation of the blood–brain barrier (BBB) disruption after cerebral I/R. Structural and functional support for the BBB is the neurovascular unit formed by endothelial cells (coded in light pink), pericytes (in green), astrocytes (in blue), and extracellular matrix components. The main physical barrier limiting paracellular diffusion between the blood and brain parenchyma is the TJ (shown as small dark red dumbbell-shaped structures on the apical side of endothelial cells). In the disruption of the BBB after cerebral I/R, the leading role is played by proinflammatory mediators, including tumor necrosis factor (TNF) and matrix metalloproteinases MMP-2/MMP-9, which are under the control of the JNK pathway. After I/R, microglia and leukocytes generate reactive oxygen species (ROS). ROS activate JNK and enhance the secretion of various cytokines, including TNF. TNF can induce ROS generation and JNK activation. ROS and JNK promote neuronal apoptosis, TJ disruption and neuroinflammation.

The BBB is a semi-permeable barrier between blood and nervous tissue that prevents large molecules, blood cells, including immune system cells, microorganisms, toxic substances and metabolites from entering the brain, and has specialized transport systems to perform this function, ensuring the transfer of compounds in both directions [58,59]. Endothelial cells, astrocytes, pericytes, and extracellular matrix components, collectively known as the neurovascular unit, provide structural and functional support for the BBB [60,61]. The neurovascular unit functions as a complex that combines vessels and astrocytes with neurons [62]. The key element of the BBB are the endothelial cells of the brain microvessels, held together by tight junctions (TJs) [63] (Figure 1). TJs consist of integral membrane proteins, such as claudins, occludin, tricellulin, and junction adhesion molecules, as well as several cytoplasmic accessory proteins [64,65]. TJ proteins, located on the apical side of endothelial cells, are the main physical barrier limiting paracellular diffusion between the blood and brain parenchyma [58].

## 4. BBB Permeability in Ischemic Stroke and Neuroinflammation

BBB disruption during ischemic stroke leads to vasogenic edema, hemorrhagic transformation, neurological failure, synaptic and neuronal dysfunction, and cognitive changes, which contribute to worsening stroke outcome [17,64,66,67,68]. The main pathological processes that damage the BBB are oxidative stress, neuroinflammation, and apoptosis. In the middle cerebral artery occlusion with reperfusion (MCAO/R) model, BBB disruptions were also observed in the contralateral hemisphere as well. These disruptions included vacuolated endothelial cells containing large autophagosomes, degenerated pericytes containing mitochondria with cristae disruption, degenerated astrocytes, perivascular edema, and parenchymal astrogliosis formation [69]. During cerebral I/R, there is a close relationship between neuroinflammation and increased BBB permeability with cerebral edema. The inflammatory cascade is activated immediately after vascular occlusion and blood flow stasis due to the release of proinflammatory mediators from the endothelium, intravascular leukocytes, and brain parenchyma [70,71].

In MCAO/R models, the activation of resident microglia and astrocytes occurs within hours after cerebral artery occlusion [72,73]. Microglia activation results in inflammasome-mediated release of interleukin 1β (IL-1β) and tumor necrosis factor (TNF) production, which participate in the inflammatory cascade by inducing cytokine and chemokine production in endothelial cells and astrocytes [74]. Loss of vascular integrity results in disruption of the BBB, which leads to increased extravasation of immune cells into the brain parenchyma [9].

After the death of damaged cells in the brain, due to the disruption of the BBB and the brain–cerebrospinal fluid barrier, new CNS antigens are released, the reaction to which and to the inflammatory mediators produced at the site of injury appears several days and weeks after the event [74]. Thus, the neuroinflammation plays a decisive role in both the acute and chronic phases of cerebral ischemia, and within a few days after, it becomes the main factor in increasing the permeability of the BBB and the formation of cerebral edema [70,71].

The major target of I/R injury is the neurovascular unit [75]. In cerebral ischemia, apoptosis of cells forming the neurovascular unit was detected [76,77]. In particular, apoptotic astrocytes and pericytes were detected in the peri-infarct region of the brain in mice with MCAO/R [78]. Damaged microvessels can attract activated leukocytes into the brain through the disrupted BBB, leading to sustained microglial activation and further tissue damage through prolonged production of proinflammatory cytokines such as TNF [79,80,81]. Activated neutrophils secrete matrix metalloproteinases (MMPs), which lead to the destruction of TJs that ensure the integrity of the BBB [9,68,82,83].

Oxidative stress plays an important role in BBB damage after ischemic stroke [84]. Brain tissue receives a sudden influx of molecular oxygen after blood reperfusion and subsequently produces excessive amounts of reactive oxygen species (ROS) via electron transfer in the mitochondria. An increase in ROS production can lead to elevated oxidative damage to biomolecules such as proteins and lipids, activate MMPs, alter TJ proteins, and ultimately result in BBB dysfunction [85].

Schematic representation of the blood–brain barrier (BBB) disruption following cerebral I/R is shown in Figure 1.

## 5. JNK Signaling and BBB Damage in Ischemic Stroke

Among the factors of brain injury during I/R, neuronal apoptosis is one of the leading factors [86]. JNKs are considered kinases that play a very important pro-apoptotic role in neuronal cells [32,87,88,89,90,91]. Activation of JNK pathway negatively impacts the function of each of the cellular elements of the neurovascular unit of the BBB [92,93,94,95]. In particular, the JNK pathway has a major influence on the apoptosis of microvascular endothelial cells and oligodendrocytes that form the neurovascular unit [96,97]. Moreover, JNK may participate in the response of endothelial cell and perivascular astrocytes to cerebral ischemia [98]. In cultured cerebral microvascular endothelial cells, hypoxia and aglycemia rapidly activate JNK [98].

**Figure 2:**
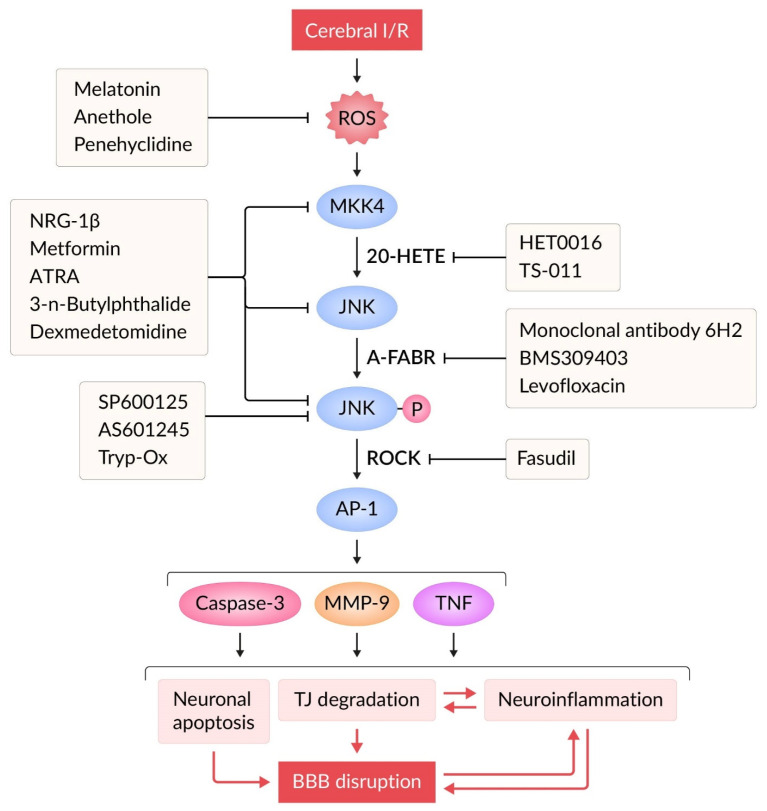
Blocking the JNK signaling pathway with JNK inhibitors and indirect biological modulators after cerebral I/R. Blocking ROS or JNK activity with JNK inhibitors restores BBB, most likely by interfering with the secretion of proinflammatory and proapoptotic mediators in cells of neurovascular unit and blocking apoptotic signaling pathways. Abbreviations: AP-1, transcription factor activator protein 1; ATRA, all-trans retinoic acid; BBB, blood–brain barrier; BMS309403, a selective A-FABP inhibitor; 20-HETE, 20-hydroxyeicosatetraenoic acid; HET0016 and TS-011, inhibitors of 20-HETE synthesis; I/R, ischemia/reperfusion; JNK, c-Jun N-terminal kinase; MKK4, mitogen-activated protein kinase kinase 4; NRG-1β, neuregulin 1β; A-FABP, fatty acid-binding protein; MMP-9, matrix metalloproteinase-9; ROCK, Rho-associated coiled-coil containing protein kinase; ROS, reactive oxygen species; TNF, tumor necrosis factor; TJ, tight junction; Tryp-Ox, tryptanthrin-6-oxime.

JNK cascade is a key pathway for cytokine release from microglia exposed to hypoxia [99,100]. During I/R, TNF activates proinflammatory gene expression and JNK-mediated apoptotic cascades. JNK activation can further stimulate TNF synthesis via activator protein 1 (AP-1) transcription factor [49] and promote microglia-mediated neuroinflammation and BBB permeability impairment [101]. ATP competitive JNK inhibitor AS601245 reduced microglial activity, IgG extravasation, activation of caspase-3 pathway in endothelial cells and oligodendrocyte progenitors and attenuated perivascular aggregation of p-JNK-positive cells 24 h after stroke [54] (Figure 2).

In I/R models, the JNK pathway has been shown to play a role in oxidative stress-induced neuronal injury [102,103]. It has also been shown that JNK, together with ERK and p38 signaling pathways, can be activated by ROS in neurons and microglia during I/R [104,105]. JNK may regulate BBB integrity through the expression of TJ proteins such as clautin-5 and zonula occludens-1 (ZO-1) [106]. In a methamphetamine-induced model of BBB injury, the JNK inhibitor SP600125 prevented the increase in MMP-9 activity and BBB disruption [107]. The authors suggest that the decrease in BBB integrity may be mediated by JNK, and this pathway activates MMP-9, causing BBB damage. A close relationship between JNK and MMPs during traumatic brain injury was demonstrated by Cheng with co-authors [108]. They showed that this injury induces an increase in S1pr2 specifically in endothelial cells, leading to MMP-9 transactivation by enhancing JNK/c-Jun signaling with following degradation of TJ proteins and increased BBB permeability. Microglia, neurons, astrocytes, and endothelial cells express Toll-like receptor 2 (TLR2) [109]. TLR2 can regulate MMP-9 expression through JNK signaling in brain microvascular endothelial cells [110].

These data suggest that the JNK pathway actively contributes to disruption of the structural integrity of the BBB in ischemic stroke. This justifies the search for new targeted approaches aimed at regulating the JNK activity to maintain the integrity of the BBB. These approaches may form the basis for new treatment strategies for I/R-induced brain injury [66].

## 6. Inhibition of JNK Signaling Pathway in Models of Ischemic Stroke

### 6.1. JNK Inhibitors

Despite many studies proving the link between activation of JNK cascade and BBB disruption, there are only a few studies on JNK inhibitors during cerebral I/R and BBB permeability. JNK inhibitor SP600125, administered together with p38 inhibitor SB203580 before MCAO/R, reduced the neurological deficits, attenuated BBB disruption and infarct volume [111]. In a rat model of MCAO/R, the JNK inhibitor SP600125 (1 mg/kg, intracerebroventricularly 30 min before MCAO/R) reduced JNK activity, decreased postischemic neuronal apoptosis, attenuated BBB disruption and infarct volume [112].

Tryptanthrin oxime (Tryp-Ox) was found to have an inhibitory effect on JNK1-3 [113]. The compound was predicted to have high BBB permeability [114]. Administration of Tryp-Ox (10 mg/kg, i.p. during the brain ischemia and then daily for 2 days) reduced BBB permeability in the left (affected) hemisphere and in the contralateral hemisphere 24 h and 48 h after the onset of reperfusion in MCAO/R model [115]. It should be noted that there is experimental data confirming the ability of the JNK inhibitors to reduce the severity of BBB disruption in other models of CNS pathology. For example, in rats with subarachnoid hemorrhage, SP600125 prevented c-Jun phosphorylation, suppressed aquaporin 1, MMP-9, and caspase-3 activation, increased the expression of claudin-5 and ZO-1, restored TJs, and attenuated BBB disruption and cerebral edema with concomitant reduction in neuronal injury and neurological deficits [106].

Pharmacological modulation of JNK pathway and BBB permeability by JNK inhibitors in different models of I/R is shown in Figure 2 and Table 1.

### 6.2. Indirect Modulators of JNK Signaling Pathway

The JNK signaling pathway is closely linked to other cellular pathways that ensure vital activity. Therefore, JNK activity can be regulated by various substances that act on various targets and signaling associated with the JNK cascade. These compounds include diverse chemical scaffolds. The structures of several small-molecule indirect modulators of the JNK pathway with their main pharmacological mechanisms and therapeutic effects in cerebral I/R models are presented in Table 2 and Table 3, respectively.

20-Hydroxyeicosatetraenoic acid (20-HETE), a metabolite of arachidonic acid, is one of the major eicosanoids in the microvascular bed and plays an important role in the regulation of cerebral blood flow, vascular tone, ion transport, cell proliferation, and inflammatory response [139,140]. 20-HETE increased vascular ROS production and promoted nuclear factor-κB (NF-κB) activation in brain microvascular endothelial cells [141]. Inhibitors of 20-HETE formation, HET0016 (N-hydroxy-N′-(4-butyl-2-methylphenyl)formamidine) and TS-011 (N-hydroxy-N′-[3-chloro-4-(4-morpholinyl)phenyl]formamidine), diminish in lesion volume after MCAO/R, and attenuate the injury-mediated reduction in cerebral blood flow in the ischemic stroke model [142,143]. The mechanism of this protective effect is associated with attenuating the oxidative stress, expression of MMP-9 and TJ proteins (claudin-5 and ZO-1), which is likely to be mediated through the suppression of JNK pathway [137] (Figure 2).

Anethole, a component compound of plant essential oils (anise, fennel, and camphor oils) is a natural antioxidant with anti-inflammatory activity [144]. Prophylactic oral administration of anethole (125 and 250 mg/kg, orally for two weeks) had a neuroprotective effect, which consisted of a decrease in infarct volume, BBB permeability, neurological deficits, and neuronal apoptosis in the cerebral cortex and hippocampus of rats after MCAO/R. The therapeutic effect of anethole was associated with a decrease in JNK activation and MMP-9/MMP-2 gene expression [132].

Penehyclidine hydrochloride (PHC), a potent cholinergic antagonist, has been shown to have anti-inflammatory and neuroprotective effects [125,126]. PHC (0.1 mg/kg, i.p. or 1 mg/kg, i.p. immediately after MCAO/R) demonstrated improvement in neurological deficits and BBB integrity, as well as reduction in infarct volume, brain water content, and neuronal apoptosis. In addition, PHC suppressed TNF, IL-1β, and ROS production and decreased the phosphorylation levels of JNK and c-Jun, indicating that the compound protects against cerebral I/R injury by downregulating the JNK cascade [136].

Melatonin is synthesized in the pineal gland and is known to have antioxidant activity [129]. Melatonin prevented TJ protein degradation and apoptosis of bEnd.3 cells, endothelial cells isolated from brain tissue derived from a mouse with endothelioma, under conditions of oxygen–glucose deprivation followed by reoxygenation (OGD/R model). In the endothelial cells, melatonin reduced the ROS production, increased the expression of TJ protein claudin 5, and attenuated JNK phosphorylation [138].

Adipocyte fatty acid-binding protein (A-FABP), an adipokine abundantly expressed in adipocytes and macrophages, plays an important role in the pathogenesis of ischemic stroke and is an independent prognostic biomarker in patients with acute ischemic stroke, closely associated with early stroke recurrence and early death [145,146,147]. A-FABP (125 and 250 mg/kg, 1 and 12 h after MCAO, and then once per day for the next six consecutive days) aggravates I/R injury in stroke by activating the JNK pathway, promoting MMP-9 expression, which causes BBB disruption [18]. A-FABP enhances inflammation by forming a positive feedback loop with the JNK cascade. A-FABP stimulates lipopolysaccharide-induced JNK phosphorylation and subsequent activation of the AP-1 transcription factor. In turn, activated JNK increases A-FABP expression by inducing phosphorylation of c-Jun, which binds to AP-1 in the A-FABP gene promoter and enhances gene transcription [148]. Anti-A-FABP monoclonal antibody 6H2 effectively neutralized A-FABP-induced JNK/c-Jun activation and reduced MMP-9 production in macrophages. In MCAO/R model in mice, 6H2 attenuated BBB disruption, cerebral edema, infarct size, neurological impairment, and mortality [131] (Figure 2, Table 3). MCAO-induced expression of A-FABP and MMP-9 in the brain was associated with a significant increase in JNK and c-Jun phosphorylation. In cerebral resident microglia, A-FABP upregulation promoted MMP-9 transactivation via potentiation of JNK/c-Jun signaling, enhancing degradation of TJ proteins and BBB disruption. JNK inhibition attenuated A-FABP-induced MMP-9 expression [18]. In this I/R model, BMS309403, a selective A-FABP inhibitor, reduced infarct volume, BBB disruption, cerebral edema, neurological deficits, and neuronal apoptosis, and increased mouse survival. In primary murine macrophages, levofloxacin inhibited A-FABP-induced JNK phosphorylation and expression of proinflammatory factors and MMP-9 [120]. In MCAO/R mouse model, levofloxacin (20 mg/kg or 60 mg/kg, i.v. for 3 days after reperfusion, once per day) attenuated BBB disruption and neuroinflammation, resulting in reduced cerebral infarction, improved neurological outcomes, and increased survival. Levofloxacin also reduced MMP-9 expression and activity and thus reduced the degradation of extracellular matrix and endothelial TJ proteins [120].

In a MCAO/R rat model, all-trans retinoic acid (ATRA) stabilized the BBB by maintaining the expression of ZO-1 and vascular endothelial cadherin [149]. ATRA (pretreated i.p. at dose of 20 mg/kg or 60 mg/kg) attenuated neurological deficits, reduced infarct volume, decreased BBB permeability, reduced TJ protein degradation, and inhibited the expression and activity of MMP-9 [149,150]. ATRA also reduced JNK phosphorylation in brain tissue. Since the protective effect of ATRA in MCAO/R can be abolished by the administration of the retinoic acid receptor α antagonist Ro41-5253, the JNK inhibitor SP600125, and the p38 inhibitor SB203580 [111], the authors suggest that the ability of ATRA to attenuate BBB disruption and the consequences of MCAO may be associated with the inhibition of the JNK and p38 signaling cascades.

Propofol is clinically used as an anesthetic, it reduces cerebral blood flow, oxygen consumption and intracranial pressure. Propofol inhibits the activation of proapoptotic genes, thereby reducing neuronal apoptosis and improving prognosis may protect tissues from cerebral I/R injury [151,152,153]. In rats with MCAO/R model, propofol (20 mg/kg/h or 40 mg/kg/h, i.v. for 30 min, 30 min following reperfusion) reduced brain tissue water and Evans blue content, and expression of MMP-9 and p-JNK in brain tissue [20].

Dexmedetomidine belongs to the group of selective D2-adrenergic agonists with central analgesic functions [118]. Administration of dexmedetomidine (9 μg/kg, i.v. 30 min after MCAO/R) reduced BBB permeability, cerebral edema, infarct volume, and neuroinflammation, suppressed JNK and MMP-9 activation, and promoted M2 microglia polarization in a rat I/R model [66].

MicroRNAs (miRNAs), a novel class of noncoding RNAs of approximately 18–22 nucleotides, participate in various fundamental biological processes, including cell proliferation, differentiation and apoptosis [67]. Elevated miR-152-3p expression in the hippocampus of mice following I/R was reported to be associated with the neuroprotection induced by postconditioning [154]. In a rat MCAO/R model, miR-152-3p (5 mL of 100 mM, 0.2 µL/min into the right lateral ventricle of rat brain, 2 h before MCAO) was shown to reduce infarct volume, attenuate neurological deficits and BBB disruption, and increase the levels of claudin-5 and occludin [133]. The authors also studied the mechanism of the protective effect of miR-152-3p in an in vitro model of BBB disruption using bEnd.3 cell monolayers under OGD/R conditions. bEnd.3 cell monolayers overexpressing miR-152-3p were protected from OGD/R-induced microvascular hyperpermeability. In turn, the protective effect of miR-152-3p was mediated by a reduction in endothelial apoptosis via a pathway associated with modulation of JNK cascade.

Fasudil, an inhibitor of Rho-associated kinases, repairs nerve damage and promotes axonal regeneration and is a potential drug for treating neurodegenerative diseases [119]. In a transient MCAO model in rats, fasudil (40 mg/kg, i.v. at 2, 4, and 24 h after MCAO/R) reduced the extent of brain injury and attenuated neurological deficits [19]. Moreover, rats treated with fasudil maintained BBB integrity, inhibited the degradation of proteins ZO-1 and occludin, and reduced MMP-9 expression and microglia-induced neuroinflammation. One of the mechanisms of the neuroprotective effect of fasudil in cerebral I/R is the inactivation of the JNK cascade, which leads to a decrease in neuroinflammation and recovery of BBB integrity [19].

Multiple molecular mechanisms of metformin include reduction in oxidative stress, endoplasmic reticulum stress, apoptosis, and protein synthesis, as well as insulin resistance in endothelial cells, cardiomyocytes, and cardiac fibroblasts through AMP-activated protein kinase (AMPK)-dependent and AMPK-independent pathways [130]. Intracerebral hemorrhage upregulates JNK3 and c-Jun phosphorylation resulting in neuron apoptosis, which can be alleviated by metformin [155]. Metformin treatment (200 mg/kg, i.p. at the time of reperfusion) reduced cerebral edema, infarct volume, and BBB disruption at 72 h after MCAO/R [134]. Pericytes plays a crucial role in regulating the permeability of the BBB [156]. Metformin treatment exerts beneficial effects on brain I/R injury by preventing pericytes apoptosis and relieving TJ loss via suppressing JNK signaling, which alleviates BBB disruption.

3-n-Butylphthalide is a neuroprotector with a multi-target mechanism of action. The potential mechanisms of 3-n-butylphthalide for ischemic stroke treatment may target different pathophysiological processes, including antioxidant, anti-inflammation, anti-apoptosis, anti-thrombosis, and protection of mitochondria [123]. The compound (per oral administration before I/R at dose of 75 mg/kg, daily for 7 days) reduced cerebral infarct volume, brain water content and BBB permeability, nerve cell apoptosis, ROS production, malondialdehyde content, and increased superoxide dismutase (SOD) activity. In addition, 3-n-butylphthalide decreased p-JNK without affecting p-ERK level, indicating that the drug administration specifically prevented the JNK activation after cerebral I/R [135].

The neuregulins (NRGs) are cell–cell signaling proteins that are ligands for receptor tyrosine kinases of the ErbB family [157]. NRG-1β, a paracrine protein released by microvascular endothelial cells, significantly improves functional recovery after focal ischemic stroke [158]. Administration of NRG-1β to MCAO/R rats (2 μg/kg, into the internal carotid artery, 2 h after MCAO) results in inhibition of MAPK kinase 4 (MKK4), JNK, and c-Jun phosphorylation, which is accompanied by restoration of BBB integrity and reduction in infarct volume [112] (Figure 2, Table 2 and Table 3).

## 7. JNK Signaling Pathway in BBB Permeability Impairment in AD

Various signaling pathways, such as Wnt/β-catenin, Notch, JNK cascade, and phosphatidylinositol 3-kinase (PI3K)/Akt/mTOR are involved in AD pathogenesis [159]. Recently, BBB dysfunction has been considered as a key pathological feature of AD [160]. Increased BBB permeability can have potentially catastrophic consequences for the neuronal homeostatic environment. Evidence of BBB impairment in AD patients is an abnormally high level of plasma proteins in cerebrospinal fluid or brain parenchyma [161,162]. A correlation has been found between the degree of BBB impairment and the stage of AD [161]. BBB disruption accelerates the progression of AD by preventing the removal of amyloid β-protein (Aβ) from the brain [163]. Increased BBB permeability has been described in animal models of AD [164,165]. In many diseases, including AD, increased BBB permeability correlates with disruption TJs [166]. Thus, morphological changes in the TJs between endothelial cells of the brain were observed in biopsies of patients with AD [167]. Pharmacological modulation of BBB permeability and JNK signaling in models of AD is summarized in Table 4.

In AD, the toxic effects of Aβ contribute to oxidative stress and chronic inflammation, disrupting the structure and function of the cerebral vascular endothelium [171]. Aβ_1–42_ reduced occludin expression and induced claudin-5 translocation from TJs in primary rat brain endothelial cells [172]. Occludin downregulation was found in APP/PS1 mice (double transgenic mice expressing a chimeric mouse/human amyloid precursor protein and a mutant human presenilin 1) [173]. Degeneration of pericytes are accompanied by BBB breakdown, aggravating brain aging in AD patients [174]. BBB disruption in AD, which is primarily due to decreased TJ protein levels and pericyte dysfunction, may accelerate neuroinflammation and structural damage to neurovascular units, leading to synaptic loss and cognitive impairment [175,176].

JNK is involved in numerous pathological processes that occur in AD. JNK3 in neurons promotes tau tangle formation by phosphorylating tau protein [177], which acts as a pathway for neurotransmitter transport [49]. Additionally, JNK3 is involved in the phosphorylation of amyloid precursor protein, leading to Aβ formation [178]. JNK3 mediates cell death through c-Jun activation and increased Fas ligand expression [179]. In AD, circulating T cells can infiltrate the brain, causing neuroinflammation [180]. This is facilitated by Aβ-dependent C-C chemokine receptor 5 (CCR5) expression in brain endothelial cells, which occurs because of JNK activation [181]. Moreover, JNK inhibition resulted in blockade of MMP2 expression, implying a direct role of JNK in the regulation of MMP2 expression and BBB permeability [182].

To assess the effect of the compounds on BBB permeability in an in vitro model, the human brain microvascular endothelial cell line hCMEC/D3 incubated with Aβ was used. Aβ_1–40_ caused a significant increase in hCMEC/D3 cell permeability to dextran, which was associated with a specific decrease in the expression of occludin protein [168]. The JNK inhibitor SP600125 prevented both Aβ_1–40_-mediated occludin downregulation and an increase in intercellular permeability in hCMEC/D3 cells. The effect of somatostatin on hCMEC/D3 permeability, JNK activation, MMP2 expression, and the level of ZO-1 and occludin proteins in cells exposed to Aβ_1–42_ was also studied [169]. The authors convincingly demonstrated that the phosphorylation status of JNK and MMP2 expression may play a crucial role in the impairment of BBB function during Aβ-induced injury. Aβ_1–42_ increased p-JNK content and MMP2 expression, and decreased ZO-1 and occludin in hCMEC/D3 cells, while the permeability of the cell layer increased. Under these conditions, somatostatin suppressed JNK activation and MMP2 expression. The protective effect of walnut (*Juglans regia* L.) extract on ZO-1 and occludin expression in brain tissue, as well as a number of other indicators, including JNK activation, was studied in mice with an AD model induced by intracerebroventricular injection of Aβ_1–42_ [170]. In this model of AD, walnut extract regulated the expression of ZO-1 and occludin associated with BBB function, reduced the expression and content of p-JNK, neuroinflammatory factors, and neuronal apoptosis, exhibited antioxidant effects, normalized mitochondrial function, and attenuated behavioral dysfunction and neurological deficits.

Thus, the results of the above studies indicate that the JNK signaling pathway may be an attractive therapeutic target for preventing BBB dysfunction in AD.

## 8. JNK Signaling Pathway in Blood–Tumor Barrier (BTB) Permeability

JNK signaling has been implicated in the regulation of tumor cell proliferation, motility, and cancer stem cell-like properties. For example, in non-small cell lung cancer, JNK signaling mediates ephrin type-A receptor 2 (EPHA2)-dependent tumor cell proliferation and motility [183]. Although direct evidence in brain tumor models is limited, it is plausible that JNK may contribute to the invasive and metastatic properties of brain tumors [184,185,186]. Elevated JNK activity has been observed in 86% of human glioma samples, correlating with enhanced tumorigenicity [186]. Mechanistically, JNK pathway drives gliomagenesis through the MKK4-JNK signaling module, which promotes glioblastoma multiforme (GBM) cell proliferation, stemness, and malignant progression [187]. This module enhances the tumorigenicity of glioma stem cells (GSCs), a subpopulation critical for tumor initiation and recurrence [187]. GSCs rely on JNK signaling for self-renewal and tumor-initiating capacity. Genetic or pharmacological inhibition of JNK reduces GSC proliferation and sphere-forming ability, indicating its essential role in maintaining the stem cell niche [188]. Furthermore, JNK facilitates GBM cell survival by hijacking neuronal Wnt signaling, depleting this niche factor from surrounding neurons and triggering neurodegeneration [189]. These findings collectively position JNK as a central driver of GBM pathogenesis. Targeting JNK in GSCs may therefore offer a therapeutic strategy to eliminate this resistant population and improve treatment outcomes. For example, modulating JNK’s interaction with cytoskeletal regulators or hypoxia-response pathways—both implicated in GBM progression—could provide targeted approaches [189,190]. However, further preclinical studies are needed to validate these approaches.

The blood–tumor barrier (BTB), a pathological counterpart of the BBB, is a critical structure in brain tumors that influences drug delivery and therapeutic outcomes [191]. Unlike the BBB, which serves as a protective shield for CNS by tightly controlling the passage of substances, the BTB is structurally and functionally altered due to the presence of malignant cells [192]. The BTB permeability is not uniform, creating a spatially heterogeneous environment that complicates drug delivery and therapeutic efficacy [193]. Structurally, the BTB exhibits disrupted TJs, increased endothelial fenestrations, and aberrant angiogenesis driven by tumor-derived cytokines and inflammatory mediators [194]. Endothelial cells in the BTB are characterized by the presence of pinocytotic vesicles, which are not typically found in the healthy BBB. These features contribute to the increased permeability of the BTB [191,195]. TJs between endothelial cells in the BTB are often disrupted or absent, further enhancing permeability [191,195]. Astrocytes, which play a crucial role in maintaining barrier integrity in the healthy BBB, are often absent or dysfunctional in the BTB, particularly in the core of the tumor [191]. Efflux transporters, such as P-glycoprotein and multidrug resistance proteins, are upregulated in the BTB and actively remove drugs from the brain parenchyma [191,196]. However, this permeability is non-uniform, with regions of the BTB retaining partial BBB-like features, creating a spatially heterogeneous barrier [193,197]. Furthermore, the BTB’s leakiness fosters edema and immune cell infiltration, contributing to tumor progression and treatment resistance [194,196].

BBB/BTB impairment plays a critical role in the progression of human brain tumors, including glioma, although the exact pathogenesis mechanism remains unclear [2]. In an in vitro co-culture model of RBE4 endothelial cells with U87 glioma cells, the levels of p-JNK and p-c-Jun were increased in RBE4 cells, while the expression of claudin-5 and ZO-1 was decreased compared with RBE4 cells cultured without U87 [23]. JNK inhibitor SP600125 could enhance the expression of claudin-5 and ZO-1 in RBE4 cells, which was inhibited by U87 glioma cells. In rats with a glioma model, JNK inhibitor SP600125 reduced the area and extent of BBB disruption.

Isolated results of pharmacological modulation of BBB permeability and JNK signaling in models of brain tumors are summarized in Table 4.

BTB impedes effective drug delivery to tumors due to its irregular permeability and upregulated efflux transporters, which actively expel chemotherapeutics [195]. Thus, when using JNK inhibitors to treat brain tumors, it is necessary to consider that the latter can restore BTB permeability, which can lead to a decrease in BTB permeability for therapeutic agents.

## 9. Limitations and Perspectives

For most of the compounds described in the review, their effect on JNK activity is one of the mechanisms of the integral neuroprotective effect in cerebral I/R models. These compounds can influence the permeability of the BBB due to the presence of other primary mechanisms (anti-inflammatory, antioxidant, anti-apoptotic, etc.), while the contribution of the effect on the BBB through JNK inhibition cannot always be identified. The use of JNK inhibitors in a few studies made it possible to determine the contribution of the JNK cascade both to the protection of the BBB during I/R and to the integral neuroprotective effect of the compounds [112]. At the same time, in several cases, the protective effect of the compounds through JNK inhibition was realized only partially [98,111]. The answer to the question of the relationship between the activation of the JNK pathway and impaired BBB permeability during I/R can be obtained by studying direct JNK inhibitors. There are preclinical studies on this topic by Ji with co-authors [112], which presented evidence of the ability of the JNK inhibitor SP600125 to reduce JNK activity and attenuate BBB disruptions when administered prophylactically into the cerebral ventricles in the MCAO/R model in rats. We believe that to finally resolve the issue of the ability of JNK inhibitors to protect the BBB during I/R, it is necessary to obtain experimental evidence of the efficacy of JNK inhibitors in a situation close to the clinical one, namely, when the test substance is administered during and/or after an episode of ischemia, indicating the timing of administration. In addition, as part of preclinical studies of compounds that are promising as a basis for the development of new drugs, it is necessary to evaluate the efficacy of systemic administration. A pilot study with JNK1-3 inhibitor Tryp-Ox was recently conducted taking these conditions into account [115].

The low selectivity of JNK inhibitors, due to their broad activity across JNK isoforms and potential off-target effects on other kinases, could contribute to a range of possible side effects [198,199]. Thus, when developing JNK inhibitors with properties of correctors of BBB permeability disorders, special attention should be paid to compounds with a selective effect on JNK3. This isoenzyme is expressed only in the brain, heart and testicles [36]. Selective inhibition of JNK3 may be relevant for reducing the death of oligodendrocytes or neuronal cells after stroke injury [97]. Theoretically, the use of a selective JNK3 inhibitor will avoid many of the side effects associated with the use of pan JNK inhibitors. Currently, substances with a selective effect on JNK3 have been described [200,201], which are of interest for further research. For example, we recently synthesized and reported substituted Tryp-Ox derivatives with a relatively high selectivity index [200]. These compounds may be useful for investigating the role of JNK3 in various models of neurodegenerative pathologies such as Alzheimer’s and Parkinson’s diseases.

Thrombolytic therapy leads to additional activation of MMPs, which is accompanied by further disruption of the BBB, while the incidence of hemorrhagic transformation increases 10-fold stage [11,12,16]. Therefore, it is important to evaluate the efficacy of JNK inhibitors for BBB protection also in the complex MCAO/R models with the introduction of tissue plasminogen activator (tPA) [202]. Such studies are not yet available.

It should be noted that JNK inhibitors may also have pleiotropic effects that enhance the protective effect on the BBB when it is disrupted. For example, some JNK inhibitors belong to the oximes and are nitric oxide (NO) donors [7,203]. NO is able to effectively remove ROS generated during hypoxia/reoxygenation of brain capillary endothelial cells, providing protection of the BBB [204].

The JNK pathway plays a complex role in brain tumor progression, with context-dependent functions in tumor initiation, maintenance, and therapeutic response. Tumor cells can disrupt the structure and function of endothelial cells of brain capillaries that form the BBB, activating the JNK pathway and changing the structure of TJs. JNK inhibitors could weaken these effects, minimizing damage to the BBB. However, these results are isolated, and this issue requires further study. It should also be considered that effective therapy of brain tumors requires sufficient permeability of chemotherapeutic agents through the BBB/BTB.

Analysis of information on the review topic allows us to outline several promising directions for further research:

1. It appears that effective correctors of BBB dysfunction could be direct/indirect JNK inhibitors and other compounds that preserve and/or restore TJ structure. This issue requires further in-depth investigation.
2. The use of thrombolytic therapy and thromboextraction during the “therapeutic window” in patients with ischemic stroke can cause reperfusion in the ischemic zone [12]. Unfortunately, post-ischemic reperfusion promotes increased oxidative stress, inflammation, BBB disruption, brain tissue edema, and hemorrhagic transformation of the ischemic focus [14,15]. The mechanistic profile of JNK pathway inhibitors demonstrates their capacity to attenuate key pathological processes in I/R injury. Thus, recanalization leads to the appearance of neutrophils in the lesion zone [205]. JNK inhibitors may limit the proinflammatory potential of neutrophils [206]. Thrombolytic therapy is accompanied by additional activation of the MMP cascade [12]. Decreased activity of the JNK pathway during reperfusion weakens the expression and activation of MMPs [19,66,131]. During the reperfusion period, cerebral edema may increase [207]. Therapeutic administration of direct and indirect JNK inhibitors (Table 1, Table 2 and Table 3) mitigates cerebral edema formation during I/R injury. However, the impact of JNK inhibitors on hemorrhagic transformation following tPA-based thrombolytic therapy remains poorly characterized. This critical gap could be addressed through experimental studies using the MCAO model in conjunction with tPA administration [202,208].
3. The contribution of BBB dysfunction in AD is underestimated. Increased BBB permeability in AD can have potentially catastrophic consequences for the homeostasis of the neural environment [209]. JNK is involved in numerous pathological processes that occur in AD. Although increased BBB permeability has been repeatedly shown in various in vivo AD models using dyes, in particular Evans blue [210,211,212], but there are no studies that have used this standardized method to confirm the involvement of the JNK signaling system in BBB regulation. Investigating JNK inhibitors as correctors of BBB dysfunction in this disease remains relevant.
4. Undoubtedly, a promising direction is the study of selective JNK3 inhibitors as agents for protecting and restoring the BBB in models of I/R, AD, and brain tumors. Research into the effects of selective JNK inhibitors on BBB permeability in AD has just begun and, given the above, may become one of the promising directions for developing an innovative drug for treating AD [201].
5. Loss of BBB integrity plays a critical role in the progression of brain tumors. In these tumors, the JNK signaling pathway is activated. However, studies on the effects of JNK inhibitors on BBB permeability remain in early stages. Furthermore, the feasibility of using JNK inhibitors to preserve BBB integrity has yet to be confirmed. It remains unclear whether mitigating BBB damage would improve outcomes in brain tumor therapy, highlighting the urgent need for interdisciplinary collaboration to resolve this critical gap in research.
6. Most JNK inhibitors lack isoform-specific targeting, as structural similarities between isoforms hinder the development of precise therapeutics [213]. Future strategies must prioritize isoform-selective designs (e.g., targeting JNK2/3 over JNK1 or JNK3 over JNK1/2) to mitigate these risks while retaining therapeutic efficacy across CNS diseases [200,214].

## 10. Conclusions

The disruption of the BBB integrity plays a key role in the pathogenesis of many diseases of the brain, such as ischemic stroke, Alzheimer’s and Parkinson’s diseases, epilepsy, traumatic brain injury, and infectious meningitis [5,22,23,24,25,38,45,46]. Activation of the JNK signaling pathway has a negative impact on the functioning of the cellular elements of the neurovascular unit, which leads to the BBB disruption. In other CNS diseases, such as vascular cognitive impairment, chronic cerebral hypoperfusion, and neuroimmunological diseases, impaired BBB permeability may be complicated by the course of the underlying disease and therapy [215,216,217]. The study of compounds that directly or indirectly inhibit the JNK pathway indicates that these agents can modify the permeability of the BBB in cerebral I/R. The main mechanisms of action of agents that attenuate damage to the BBB are antioxidant action, anti-inflammatory action, and reduction in MMP expression [17]. Inhibition of JNK pathway can suppress neuroinflammation, restore BBB integrity, improve myelin regeneration, and accelerate autophagic activity, which improves neuronal survival [218].

Despite the differences in the pathogenesis of AD and ischemic stroke, the mechanisms of BBB damage in these diseases are common and include neuroinflammation, oxidative stress, activation of MMPs, impaired expression and translocation of TJ proteins, and activation of numerous intracellular signaling pathways, including JNK. The results of several studies indicate that the inhibition of the JNK pathway can prevent the formation of BBB dysfunction in AD.

The role of JNK signaling in BBB disruption in brain tumors has received limited research attention. Understanding the specific contributions of JNK to brain tumor progression is essential for the development of effective therapeutic strategies that can improve outcomes for patients with brain tumors. Dual JNK functionality necessitates a precision medicine approach, wherein therapeutic interventions are tailored to the genetic and molecular context of individual tumors. Some of the compounds mentioned in the review reduce the BBB damage and may form the basis for the development of promising neuroprotectors, one of the mechanisms of action of which will be inhibition of the JNK signaling cascade. We conclude that future studies should aim to define specific isoform roles of JNK in cerebral I/R injury and neurodegenerative diseases and stratify brain tumor patients likely to benefit from JNK-targeted therapy.

## Figures and Tables

**Table 1:** Pharmacological modulation of BBB permeability by JNK inhibitors in MCAO/R models (in rats).

Inhibitor	Dosage	Biological Effect	Ref.
SP600125/SB203580	0.3 mg/0.3 mg, i.c.v.	↓ BBB permeability, brain water content, infarct volume, neurological deficits	[111]
SP600125	1 mg/kg, i.c.v.	↓ BBB permeability, infarct volume, neuronal apoptosis, JNK activity, p-c-Jun	[112]
Tryp-Ox	10 mg/kg, i.p.	↓ BBB permeability	[115]

Abbreviations: Tryp-Ox, tryptanthrin-6-oxime; i.c.v., intracerebroventricularly; i.p., intraperitoneally.

**Table 2:** Main pharmacological mechanisms of small-molecule compounds with reported indirect effect on JNK signaling pathway in MCAO/R and in vitro models of I/R.

Common Name	Chemical Structure	Chemical Class/Main Pharmacological Mechanism	Ref.
Anethol	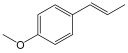	Phenylpropanoid with antimicrobial and immunomodulate activity.	[116,117]
Dexmedetomidine	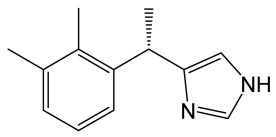	Imidazole derivative with analgesic and sedative properties, agonist of α_2_-adrenergic receptors.	[118]
Fasudil	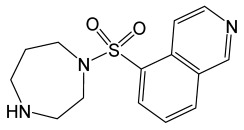	Isoquinoline derivative, potent Rho-kinase inhibitor and vasodilator.	[119]
Levofloxacin	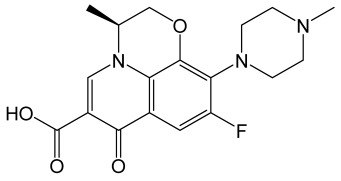	Broad-spectrum, third-generation fluoroquinolone antibiotic used to treat bacterial infections, displayed A-FABP inhibitory activities.	[120,121]
All-trans retinoic acid	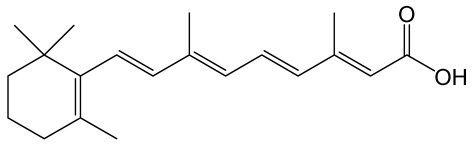	The vitamin A derivative binds to two nuclear receptors in keratinocytes: retinoic acid receptor and retinoid X receptor.	[122]
3-n-Butylphthalide	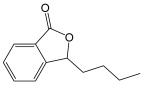	Benzofuran derivative, an inhibitor of TWIK-related expressing K^+^ channel 1(TREK-1) with antioxidant, anti-inflammation, and anti-apoptosis activities.	[123,124]
Penehyclidine hydrochloride	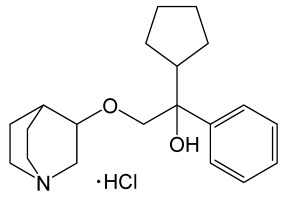	Quinuclidine compound, an anticholinergic agent, a selective antagonist of muscarinic M1 and M3 acetylcholine receptors, with anti-inflammation and neuroprotection effects.	[125,126]
Propofol	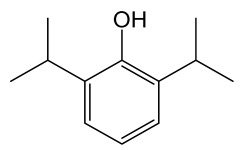	Phenol derivate with anesthetic effect; potentiates the inhibitory effects of the neurotransmitter gamma-aminobutyric acid (GABA) by binding to and activating GABA receptors in the CNS.	[127]
HET0016	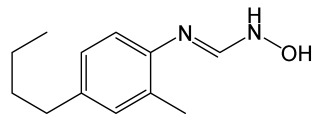	N-hydroxy-N’-(4-butyl-2-methylphenyl)-formamidine, a potent and selective 20-HETE synthase inhibitor.	[128]
Melatonin	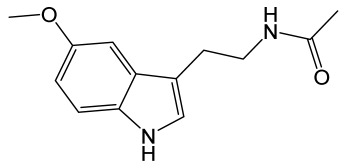	Derivative of serotonin, a hormone, which regulates the body’s sleep–wake cycles by interacting with the suprachiasmatic nucleus of the hypothalamus and the retina.	[129]
Metformin	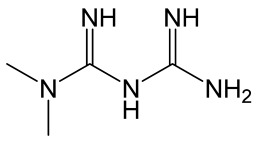	Biguanide derivative. Reduces glucose production in the liver by inhibiting the enzyme complex I in the mitochondria with following AMPK (AMP-activated protein kinase) activation.	[130]

**Table 3:** Modulation of BBB permeability by indirect inhibitors of JNK pathway in MCAO/R and in vitro models of I/R.

Substance	Animals (Model)/In Vitro (Cells)	Dosage/Concentration	Biological Effect	Ref.
Dexmedetomidine	Rats (MCAO/R)	9 µg/kg, i.v.	↓ BBB permeability, neuroinflammation, MMP-9 level, JNK and p38 signaling	[66]
Antibodies against A-FABP	Mice (MCAO/R)	1.8 and 3.6 mg/kg	↓ BBB permeability, cerebral edema, infarct volume, neurological deficits, mortality, MMP-9, p-JNK, p-c-Jun	[131]
	In vitro (Mϕ)	1 μg/mL	↓ MMP-9 level and p-JNK	[131]
A-FABP inhibitor	Mice (MCAO/R)	15 mg/kg, p.o.	↓ BBB permeability, cerebral edema, infarct volume, neuronal apoptosis, neurological deficits, mortality, JNK/c-Jun signaling; ↑ occludin and ZO-1 expression in brain tissue	[18]
Levofloxacin	Mice (MCAO/R)	30 mg/kg, i.v.	↓ BBB permeability, neuroinflammation, neurological deficits, mortality	[120]
	In vitro (Mϕ)	30 μM	↓ A-FABP-induced JNK activity	[120]
Anethole	Rats (MCAO/R)	125 and 250 mg/kg, p.o.	↓ BBB permeability, cerebral edema, neurological deficits, neuroinflammation, MMP-9, TNF, IL-6, IL-1β, and NF-κB levels, JNK and p38 signaling	[132]
miR-152-3p	Rats (MCAO/R)	5 mL of 100 mM solution, i.v.	↓ BBB permeability, infarct volume, neurological deficits; ↑ claudin-5 and occludin expression	[133]
	In vitro (bEnd.3 cells)	N.S.	↓ Apoptosis, JNK activity	[133]
Fasudil	Rats (MCAO/R)	40 mg/kg, i.v.	↓ BBB permeability, ischemic volume, neurological deficits, neuroinflammation, MMP-9 level, JNK and p38 signaling; ↑ ZO-1 and occludin expression	[19]
Metformin	Mice (MCAO/R)	200 mg/kg, i.p.	↓ BBB permeability, cerebral edema, neurological deficits, apoptosis of pericytes, JNK and p38 activation; ↑ neoneurogenesis	[134]
All-trans retinoic acid	Rats (MCAO/R)	10 and 30 mg/kg, i.p.	↓ BBB permeability, ischemic volume, neurological deficits, degradation of TJ proteins, MMP-9 expression and activity, JNK and p38 activity	[111]
3-n-Butylphthalide	Rats (MCAO/R)	75 mg/kg, p.o.	↓ BBB permeability, cerebral edema, infarct volume, neuronal apoptosis, ROS production, MDA, JNK and p38 activation; ↑ SOD activity	[135]
Penehyclidine hydrochloride	Mice (MCAO/R)	0.1 and 1 mg/kg, i.p.	↓ BBB permeability, brain edema, neurological deficits, infarct volume, neuronal apoptosis, ROS production, TNF, IL-1β, p-JNK, p-p38, p-c-Jun; ↑ SOD and GSH-Px activity	[136]
Propofol	Rats (MCAO/R)	20–40 mg/kg/h, i.v.	↓ BBB permeability, cerebral edema, aquaporin-4/MMP-9-positive cells, JNK activity	[20]
HET0016	Rats (MCAO/R)	1 mg/kg, i.v.	↓ BBB permeability, MMP-9, JNK and c-Jun activation; ↑ claudin-5 and ZO-1 expression	[137]
Neuregulin-1β	Rats (MCAO/R)	2 μg/kg, i.c.	↓ BBB permeability, infarct volume, neurological deficits, neuronal apoptosis, p-MMK4, p-JNK, p-c-Jun	[112]
Melatonin	OGD/R (in vitro, bEnd.3 cells)	10 and 100 nM	↓ ROS production, p-JNK; ↑ claudin-5 expression	[138]

Abbreviations: Mϕ, macrophages; MCAO/R, middle cerebral artery occlusion/reperfusion; A-FABP, adipocyte fatty acid-binding protein; GSH-Px, glutathione peroxidase; MDA, malondialdehyde; MMP, matrix metalloproteinase; i.v., intravenously; i.c., into the internal carotid artery; p.o., per oral; OGD/R, oxygen–glucose deprivation/reoxygenation; ROS, reactive oxygen species; SOD, superoxide dismutase; ZO-1, zona occludens-1; N.S., concentration not specified.

**Table 4:** Pharmacological modulation of BBB permeability and JNK signaling in models of AD and brain tumor.

Substance	Model	Concentration/Dosage	Biological Effect	Ref.
Models of Alzheimer’s Disease				
SP600125	hCMEC/D3 cells, Aβ_1–40_	50 µM	↓ intercellular permeability in hCMEC/D3 cells↑ occludin expression	[168]
Somatostatin	hCMEC/D3 cells, Aβ_1–42_	0.4–10 µM	↓ intercellular permeability in hCMEC/D3 cells; p-JNK and MMP-2	[169]
*J. regia* L. extract	Mice, Aβ_1–40_ i.c.v.	20 mg/kg	↓ p-JNK↑ claudin-5 and ZO-1 expression	[170]
Models of Brain Tumor				
SP600125	RBE4 with U87 cells (co-culture, in vitro model)	N.S.	↑ claudin-5 and ZO-1 expression	[23]
	Rats (glioblastoma, in vivo model)	N.S.	↓ BBB permeability	

N.S., concentration not specified; i.c.v., intracerebroventricularly.
